# History of a Case in Which Hydatids Were Voided by the Mouth, and Likewise from a Large Tumor on the Back

**Published:** 1785

**Authors:** 


V. *Hiſtory of a Caſe in which Hydatids were voided by the Mouth, and likewiſe from a large Tumor on the Back. By Mr.* J. Powell, *Surgeon at Brecon. Communicated in a Letter to* William Oſborn, M. D. *and by him to Dr.* Simmons.

A YEOMAN of the pariſh of Llandſputhet, in the county of Brecon, conſulted me a few years ago, about a large tumor, of the encyſted kind, ſituated on the back, and extending from the ſuperior part of the right ſcapula quite to the ilium of the ſame ſide.

Upon making an incifion the whole length of the tumour, between five and fix quarts of indigefted matter were difcharged, with an inumerable quantity of hydatids fwimming in it. The wound appeared tolerably well for fome weeks, but the difcharge was fo great, that the health and ftrength of my patient gradually declined; and a cough and hectic fever coming on, feemed to threaten a fpeedy and fatal conclufion to all his fufferings, when nature firft pointed out that means of relief, which art feems to have had no inconfiderable fhare in accomplifhing.

About a month after the tumour had been opened, he one day complained of a moft difagreeable and naufeous tafte, for which I advifed, and he took, a common emetic. This operating in the ufual manner, produced no other immediate or fenfible effect; but foon afterwards, he was feized with fo violent a rigor, fucceeded by fuch cold clammy fweats, and accompanied with a countenance fo ghaftly, that upon my firft feeing him, he appeared to be in a dying ftate; but prefently, a difcharge of matter, apparently of the fame kind as that which had been difcharged from the tumour, iffued from his mouth, and continued flowing for for more than two hours; his countenance now recovered its natural appearance, and from that period all his ſymptoms began to abate; his cough ceaſed, and his hectic fever left him, inſomuch, that with the aſſiſtance of the bark, and a milk diet, in ſix weeks he was perfectly recovered.

